# Effects of Drought on the Water Use Strategies of Pure and Mixed Shrubs in the Mu Us Sandy Land

**DOI:** 10.3390/plants13233261

**Published:** 2024-11-21

**Authors:** Qin Gao, Xiaohong Dang, Zhongju Meng, Yang Liu, Jiale Lou, Yu Yan, Xing Zhang

**Affiliations:** 1 College of Desert Control Science and Engineering, Inner Mongolia Agricultural University, Hohhot 010018, China; gaoqin0823@126.com (Q.G.);; 2 Inner Mongolia Hangjin Desert Ecosystem National Positional Research Station, Ordos 017400, China; 3 Inner Mongolia Autonomous Region Water Resources Research Institute, Hohhot 010018, China

**Keywords:** stable isotope, pure shrubs, mixed shrubs, water use strategy, Mu Us Sandy Land

## Abstract

Water resources are crucial factors that limit vegetation recovery, and rational planning of silvicultural patterns is essential for the efficient utilization of water in arid and semi-arid regions. This study examined the water utilization strategies of pure shrubs (pure stands of *Artemisia ordosica* and pure stands of *Salix psammophila*) and mixed shrubs (mixed stands of *A. ordosica S. psammophila*, and mixed stands of *A. ordosica Caragana korshinskii*) from the rainy to dry seasons using stable isotope techniques and MixSIAR modeling in the Mu Us Sandy Land in the semi-arid region of China. Mixed shrubs were significantly more effective than pure shrubs in utilizing the primary water sypply from the soil layer. During the rainy season in August, shallow soil water was used to a greater extent, contributing 33.78 ± 2.18%, with no significant difference in the contribution proportion. After a brief drought during the transition period in September, there was a significant increase in the use of the primary water-absorbing soil layer across all vegetation types, with a maximum increase of 39.53%. Conversely, during the dry season in October, after an extended drought, the contribution of the primary water supply layer to vegetation water absorption decreased compared with the transition period, with a maximum increase of only 17.88%. The results of this study revealed that variations in water conditions and vegetation configurations influence the water utilization patterns of the vegetation. This study offers a scientific basis and theoretical support for understanding ecological water use, the rationale behind vegetation establishment, and an assessment of plantation community stability in sandy regions.

## 1. Introduction

One of the most significant ecological and environmental issues is the inadequate water supply, which has garnered considerable attention [1]. Water is a crucial ecological factor constraining vegetation distribution and growth in arid and semiarid regions with limited precipitation. Water scarcity directly alters the composition and distribution of the plant communities [2]. Mu Us Sandy Land, a typical semi-arid region, has experienced a significant decline in vegetation cover due to unsustainable practices, including overgrazing and careless land reclamation in earlier years. Large-scale vegetation restoration initiatives have substantially improved this over the past 50 years [3]. Over the past 30 years, the regional NDVI has shown a significant upward trend, indicating an increase in vegetation cover of nearly 17.65% [4]. However, the scale and density of forest planting were not adequately considered during the planning process, resulting in an imbalance between water supply, primarily from precipitation, and water consumption through evapotranspiration [5]. In the context of ongoing afforestation initiatives and increasing water scarcity, it is imperative to optimize ecological functions and adopt a “water-centric planting” approach. This strategy aims to enhance the quality of afforestation efforts and develop systems that align with the local water resource thresholds. Sandy shrubs are vital vegetation types for achieving sand stabilization and revegetation projects in arid and semi-arid regions. Understanding the water use strategies of sandy shrubs is essential for modeling and analyzing hydrological processes at the soil-vegetation-atmosphere interface, and for gaining valuable insights into the adaptability of vegetation to environmental changes [6].

Plant water utilization can reveal the survival strategies and responses of plants to environmental changes, which is critical for understanding their adaptation and tolerance to drought stress [7]. Research has shown that vegetation adapts to arid environments through various water-use strategies [8,9]. However, findings regarding the correlation between drought tolerance and water-use strategies are inconsistent. Some studies have indicated that vegetation utilizing shallow soil water exhibits high drought tolerance, whereas those relying on deeper soil water tend to be less drought-tolerant [10]. Additionally, it has been suggested that vegetation that taps deeper into the soil water demonstrates better drought adaptation when shallow soil water availability is diminished [11]. Localized disturbances in environmental conditions and vegetation traits can also hinder the effectiveness of self-regulation among plant communities [12]. This phenomenon is particularly pronounced in pure-community plantations characterized by monoculture stand structures [13]. In contrast, mixed communities optimize water-use patterns by reducing competition for water through ecological niche complementarity among species [14]. To some extent, competition for water among the coexisting vegetation types drives community development [15]. Previous studies on mixed forests have often focused on enhancing vegetation properties, such as increasing productivity [16], increasing carbon sinks and ecological benefits [17], reducing drought sensitivity [18], and facilitating recovery from drought [19]. Most studies on strategies for utilizing water in mixed forest vegetation begin with an examination of evapotranspiration, water transport, and water deficits [20,21] to illustrate the various ways in which different vegetation types can either facilitate or compete with one another in their relationships with soil and water. There is ongoing debate among academics regarding the depletion of vegetated water. Some researchers have suggested that vegetation water depletion varies minimally between different vegetation types [20], whereas others have argued that it is significantly higher in pure forests than in mixed forests [22]. Zhang et al. observed that two coexisting shrubs exhibited a highly adaptive utilization pattern, using shallow and deep soil water rather than competing for resources [23]. Similarly, Wang et al. found comparable results in a correlation study of mixed forests composed of three deciduous tree species in the Loess Plateau [24]. However, some researchers have observed that these two types of mixed-species vegetation primarily co-absorb shallow soil water, with the depth of absorption gradually shifting to deeper soil water after a decrease in rainfall [25]. In summary, plant-water strategies are influenced by factors such as arid environments [7] and vegetation types [26], and their differentiation and identification are highly complex and variable.

Current research on water-use strategies in arid and semi-arid regions has provided valuable insights [24,25]. Numerous experiments have been conducted to analyze changes in environmental conditions, such as seasonal rainfall and configuration patterns, in pure and mixed forests on a case-by-case basis. However, the mechanisms underlying the differences in vegetation water utilization strategies influenced by the combined effects of arid environments and vegetation types are not yet clear. In light of this, we conducted an experiment in a typical Mu Us Sandy Land area, selecting both pure shrubs (*A. ordosica*, *S. psammophila*) and mixed shrubs (*A. ordosica* and *S. psammophila*, *A. ordosica* and *C. korshinskii*). Using stable isotope techniques and MixSIAR modeling, our objectives were to (1) investigate changes in soil moisture, isotopic composition, and their vertical gradients along the soil profiles for both pure and mixed shrubs during the rainy and dry seasons in the Mu Us Sandy Land, (2) quantify changes and variations in water use sources among different silvicultural patterns from the rainy to dry seasons, and (3) uncover vegetation water use strategies under two-factor disturbances. This study enhances our understanding of the regional relationships between soil moisture and plant life as well as the ecological adaptations of artificial plants in arid and semi-arid regions with limited water resources. Additionally, it can serve as a guide for planting specifications and patterns in areas lacking vegetation restoration efforts, helping prevent the overuse of water and ensuring ecosystem sustainability during the restoration process.

## 2. Results

### 2.1. Meteorological Factors and Isotopic Composition of Rainwater

**Figure 1:**
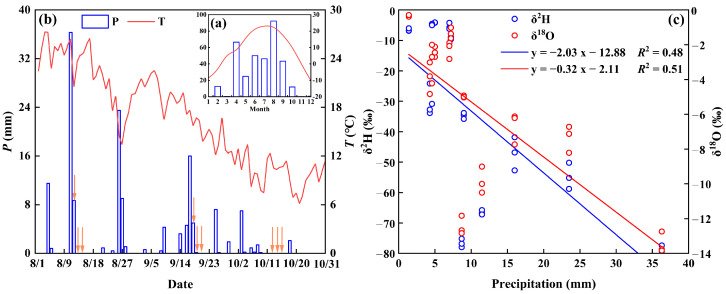
Mean monthly temperature and precipitation for 2023 in the study area (**a**), temperature and precipitation in the study area during the experimental period (**b**) (orange arrows indicate when soil vegetation samples were collected), rainfall δ^2^H and δ^18^O variations, and the linear relationship between precipitation and δ^2^H and δ^18^O (**c**).

The monthly average meteorological changes for 2023 (Figure 1a) indicate a transition from the rainy to the dry season. The cumulative local precipitation reached 348.8 mm, whereas the precipitation during the growing season (May–October) reached 268.7 mm, representing 77.04% of the annual total. August recorded the highest average precipitation of 92.2 mm, constituting 26.43% of the annual total (Figure 1a). The peak average daily temperature of 27.29 °C was recorded on 3 August during fluctuations in the average daily temperature. Between 4 August and 27 August, intermittent rainfall mitigated high-temperature conditions to some degree, with a gradual decline in temperature commencing on 21 August, ultimately reaching a minimum average daily temperature of 6.17 °C on 21 October (Figure 1b). The transition from the rainy to the dry season was clearly observed throughout the experimental period. Throughout the study period, the δ^2^H and δ^18^O values of rainfall varied from −80.43‰ to −4.08‰ (weighted average: −35.66‰) and from −13.85‰ to −0.29‰ (weighted average: −5.67‰), respectively. There was a negative correlation between rainfall isotope ratios (δ^2^H and δ^18^O) and precipitation (Figure 1c), with regression line slopes of −2.03 for δ^2^H and −0.32 for δ^18^O, indicating a diluting effect of rainfall. This indicates that hydrogen and oxygen isotopes were strongly correlated with rainfall trends.

### 2.2. Isotopic Composition of Rainwater, Soil Water, and Plant Xylem Water

**Figure 2:**
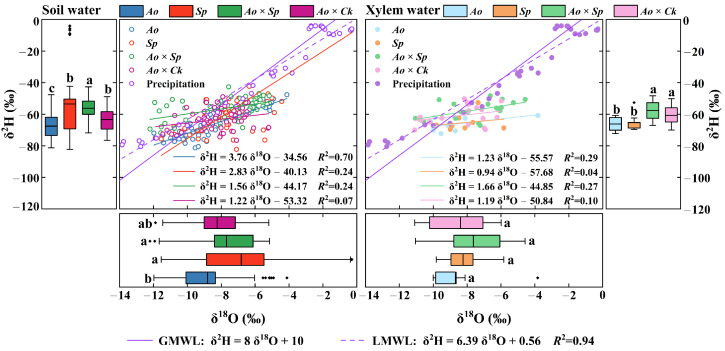
Scatterplots and histograms of δ^2^H and δ^18^Ofor rainwater, soil water, and xylem water in shrub-pure and mixed forests during the measurement period. The boxplot shows the mean (black line), interquartile range (box range), overall range (whisker line), and outliers (black dots). LMWL and GMWL represent the local and global meteoric water lines, respectively. Lowercase letters within the boxes indicate significant differences in δ^2^H and δ^18^O for soil water and xylem water across different silvicultural patterns (*p* < 0.05).

The δ^2^H and δ^18^O values from local atmospheric precipitation water samples were used to establish the LMWL, resulting in the following equation: δ^2^H = 6.39 δ^18^O + 0.56 (*R*^2^ = 0.94). The slope and intercept of the LMWL were lower than those of the global meteoric water line (GMWL), indicating that isotopic enrichment in rainfall was influenced by evaporation during the precipitation period (Figure 2). The hydrogen and oxygen isotopes of soil water and xylem water beneath various forest types were positioned on the lower right side of the LMWL, suggesting that precipitation recharged the soil water relative to xylem water, whereas evaporation affected soil water. Linear regressions of hydrogen and oxygen isotopes in soil and xylem water were conducted for each plantation. Soil water regression exhibited a steeper slope in pure forests than in mixed forests (regression slopes ranging from 1.22 to 3.76). In contrast, xylem water regression showed a steeper slope in mixed forests than in pure forests (regression slopes ranging from 0.94 to 1.66).

The isotopic values of soil moisture varied according to sampling period and vegetation type (Figure 2). The mean δ^2^H and δ^18^O isotope values of soil water in *Ao, Sp, Ao × Sp, and Ao × Ck* were −67.59‰ (CV = 12%) and −8.79‰ (CV = 21%), for *Ao*: −62.21‰ (CV = 15%) and −7.80‰ (CV = 21% for *Sp*), −56.18‰ (CV = 12%) and −7.68‰ (CV = 27%) for *Ao* × *Sp*, and−63.40‰ (CV = 11%) and −8.24‰ (CV = 18%) for *Ao* × *Ck*, respectively. A trend of depletion in δ^2^H and δ^18^O soil water isotope values was observed between *A. ordosica* and *S. psammophila* in pure forests compared with mixed forests, with the mean δ^2^H and δ^18^O soil water isotope values of being lower than those of *Ao* × *Ck*. Significant differences were observed between the pure and mixed forests (*p* < 0.05). The isotopic values of xylem moisture varied according to sampling period and vegetation type (Figure 2). The δ^2^H and δ^18^O isotope values were higher in the mixed forests of *Ao* × *Sp*, with fluctuations ranging from −67.09‰ to −48.36‰ (mean value: −57.49‰) and from −11.05‰ to −4.58‰ (mean value: −7.63‰), respectively. The δ^2^H and δ^18^O isotope values of the remaining plantation forests were ranked as follows: *Sp* (mean values: −65.39‰ and −8.24‰, respectively) > *Ao* × *Ck* (mean values: −60.80‰ and −8.38‰, respectively) > *Ao* (mean values: −66.19‰ and −8.65‰, respectively). Overall, hydroxide isotopes were influenced by the presence of multiple vegetation types in mixed forests, exhibiting a trend of depletion compared with pure forests.

### 2.3. Changes in Soil Moisture, Hydrogen, and Oxygen Isotopes During the Rainy and Dry Seasons

**Figure 3:**
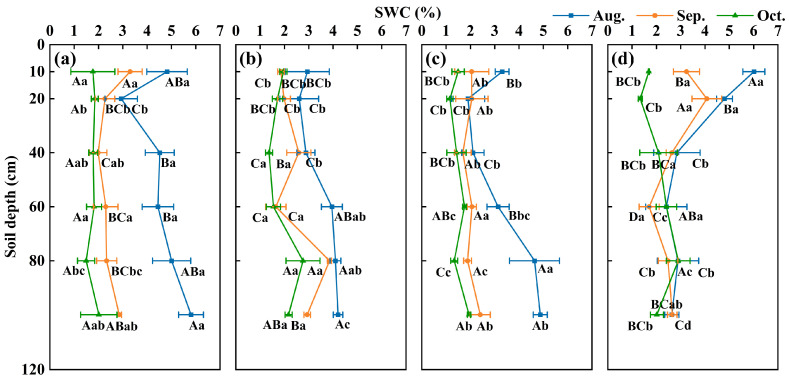
Vertical change pattern of soil water content in different plantation forests from the rainy to dry season (0–120 cm) ((**a**–**d**) represent *Ao, Sp, Ao* × *Sp,* and *Ao* × *Ck*, respectively). Capital letters indicate significant differences between the soil depths under the same vegetation cover in the same month (*p* < 0.05). Lowercase letters indicate significant differences in vegetation cover at the same soil depth during the same month (*p* < 0.05). Error bars represent standard deviation (mean ± SD, *n* = 3).

The monthly average soil water content change pattern in the 0–120 cm soil layer across the four plantation sites from the rainy to dry season is shown in Figure 3. The soil water content under different plantation forest cover types exhibited a monthly decline. This changing pattern was more closely aligned with the precipitation trend, and the soil water content in the pure communities was significantly higher than that in the mixed communities (*p* < 0.05). During the rainy season and transition period, the soil water content in the shallow soil layer of each plantation decreased with increasing soil depth, ranging from1.03% to 6.52%. In the middle and deep soil layers (20–120 cm), soil water content was influenced by the root system characteristics of *C. korshinskii* and soil water-holding capacity, showing minor differences and an insignificant pattern of change in *Ao* × *Ck* (1.23% to 3.89%). In contrast, the remaining plantation forests exhibited an increase in soil water content with increasing soil depth (1.16% to 6.11%), with some soils showing significant differences (*p* < 0.05). Field observations indicated almost no rainfall in the experimental area during the dry season. Most vegetation withered because of temperature effects, and the soil water content fluctuated less with soil layer depth. During the sampling period, the shallow soil water content displayed high variability (CV = 49%) compared with the other soil layers, which gradually stabilized with increasing soil depth (4% < CV < 31%).

**Figure 4:**
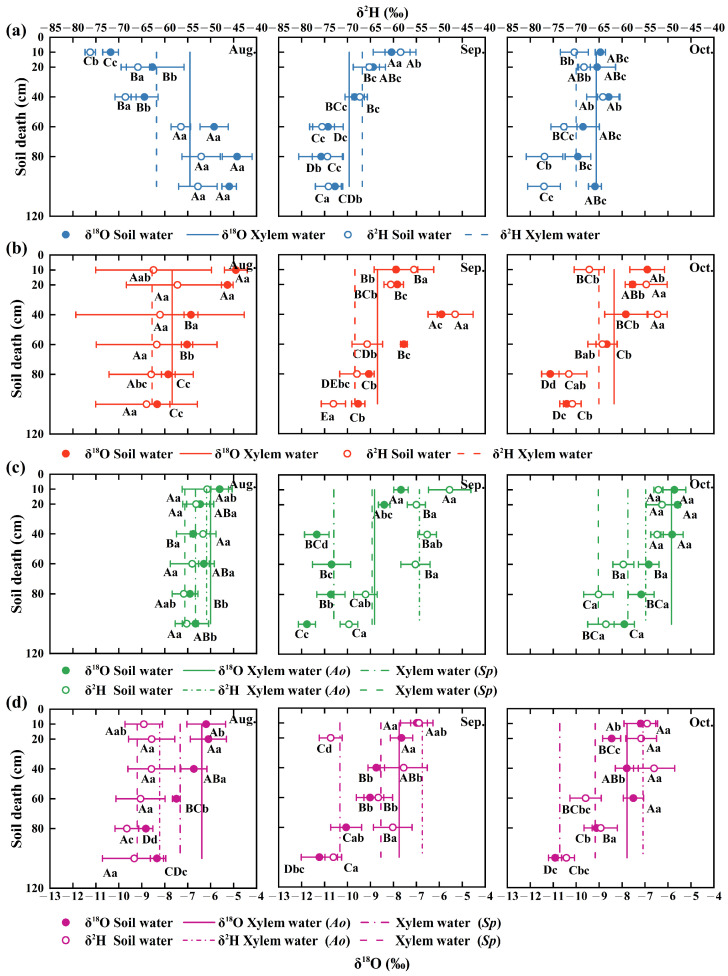
Distribution patterns of soil water and vegetation xylem water δ^2^H and δ^18^O values in different plantation forests during the experimental period with changes in soil depth and sampling time ((**a**–**d**) represent *Ao, Sp, Ao × Sp,* and *Ao* × *Ck*, respectively). Capital letters indicate significant differences between the soil depths under the same vegetation cover in the same month (*p* < 0.05). Lowercase letters indicate significant differences in vegetation cover at the same soil depth in the same month (*p* < 0.05). Error bars represent standard deviation (mean ± SD, *n* = 3).

The vertical distributions of soil water hydroxide isotope values were similar during the rainy and dry seasons in both pure shrub and mixed shrub communities. Both types exhibited a significant variation pattern concerning soil depth and stand type (*p* < 0.05) (Figure 4). In the four plantation forests, soil water isotopes were depleted layer by layer (*p* < 0.05) as soil depth increased throughout the sampling period. The δ^2^H and δ^18^O isotope values of soil water in different plantation forests were most enriched during the rainy season and most depleted during the transition period, which is associated with rainfall variability and increased plant water demand. The rain-dry transition values for δ^2^H (−59.92 ± 8.42‰ [CV = 14%]) and δ^18^O (−7.34 ± 1.46‰ [CV = 20%]) in shallow soil water were significant. *A. ordosica* was significantly enriched in mixed forests compared with pure forests by 25.09% (*Ao* × *Sp*) and 15.28% (*Ao* × *Ck*) (*p* < 0.05), respectively. The difference between *Sp* and *Ao* × *Sp* was not statistically significant (*p* > 0.05). The alternation between rain and drought in δ^2^H (−60.51 ± 8.57‰ [CV = 14%]) and δ^18^O (−8.02 ± 1.64‰ [CV = 20%]) values in mesocosmic soil water remained evident. The pattern of differences between pure and mixed forests mirrored that of shallow soil. The δ^2^H (−66.69 ± 8.04‰ [CV = 12%]) and δ^18^O (−9.03 ± 1.91‰ [CV = 21%]) values of the deep soil water were significantly less affected by the alternation of rain and drought. Soil water δ^2^H and δ^18^O isotope values were significantly higher in pure shrubs than in mixed shrubs (*p* < 0.05), with a 5.36% enrichment in *Ao* compared with the two mixed modes, and a significant increase of 8.85% in *Sp* compared with *Ao* × *Sp*. These findings illustrate that different types of plantation forests influence soil water depletion and isotope enrichment, with mixed forests demonstrating greater variability in metrics characterizing water use.

### 2.4. Water Utilization Relationship Between Pure and Mixed Forests During the Rainy and Dry Seasons

Comparison of δ^18^O levels in xylem and soil water using a direct judgment approach (Figure 4). *A. ordosica* exhibited an upward shift in the source of vegetation water use to the shallow soil layer as rainfall decreased in both pure and mixed forests. In contrast, *S. psammophila* and *C. korshinskii* in pure and mixed forests showed a decline in water use with decreasing rainfall, resulting in a downward shift in the water source to deeper soil layers.

**Figure 5:**
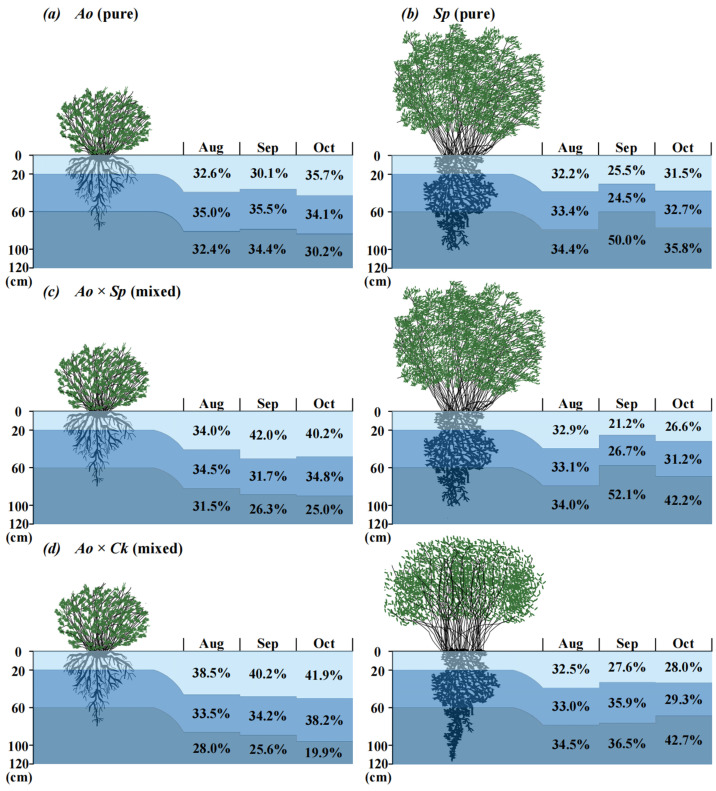
Month-to-month variation in water absorption ratio between pure and mixed shrubs in different soil layers based on MixSIAR modeling.

MixSIAR model predictions quantified the proportion of water uptake from each soil profile layer (Figure 5) within the stands. The proportions of the three potential soil water sources varied slightly and were influenced by the vegetation type and rainfall. The rainy season was characterized by precipitation events, and the variation in contributions among the soil layers was minimal. During this season, *A. ordosica* in the two mixed forests predominantly absorbed soil water from the shallow and middle layers (0–60 cm), contributing 68.5% (*Ao* × *Sp*) and 72.0% (*Ao* × *Ck*). In contrast, *S. psammophila* and *C. korshinskii* primarily absorbed water from deeper soil layers, contributing 34.0% and 34.5%, respectively. The water contribution rates of each forest site during the transition period exhibited considerable variation, with the two mixed forests of *A. ordosica* primarily absorbing shallow soil water, yielding contribution rates of 42.0% (*Ao* × *Sp*) and 40.2% (*Ao* × *Ck*). *S. psammophila*, with a contribution rate of 52.1%, and *C. korshinskii*, with a contribution rate of 36.5%, primarily absorbed deep soil water. The increase in *A. ordosica* within the mixed forests was significant, showing an increase of 39.53% (*Ao* × *Sp*) and 33.55% (*Ao* × *Ck*) in its contribution to *Ao* (which mainly involved shallow soil water). During the dry season, the water contribution of each stand remained unaffected by the rainfall. In the pure forest, *A. ordosica* predominantly absorbed shallow soil water (35.7%). *Sp* primarily absorbed deep soil water (35.8%), which represented a decrease of 12.61% (*A. ordosica*) and 17.88% (*S. psammophila*) compared with that of *Ao* × *Sp*; and a decrease of 17.37% compared to *Ao* × *Ck*.

In summary, moisture conditions and vegetation type influence the sources of water utilization, resulting in varying reflections. When moisture conditions are constant, the contribution of the main soil layer to vegetation moisture is enhanced to a certain extent in mixed forests compared with that in pure forests. When the vegetation types are identical, the source of water utilization during the rainy season is primarily concentrated in the soil surface layer, which is less affected by plant species. As rainfall recharge decreases, the sources of water utilization among different planted forests shift according to the characteristics of their root system distribution.

## 3. Materials and Methods

### 3.1. Study Area

**Figure 6:**
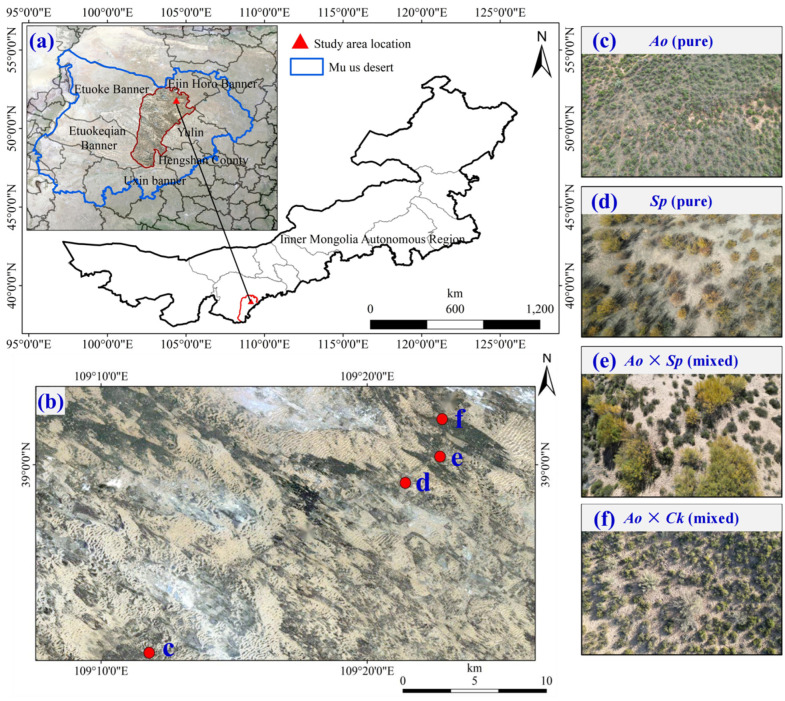
A thematic map of the study area was prepared based on Landsat 8 imagery. The blue line on the map shows the boundary line of Mu Us Sandy Land, and the red triangle shows the main study area (**a**). The red dots in the figure show the distribution of sample locations in the study area (**b**). Examples of four iconic sample plots (**c**–**f**).

The study was conducted in the hinterland of Mu Us Sandy Land in northern China (38°53′13″ N–39°01′39″ N, 109°11′49″ E–111°22′49″ E) (Figure 6), specifically in Tuke Town, Uxing Banner, Ordos City, Inner Mongolia Autonomous Region. The area is characterized by flat terrain and an elevation ranging from 1275 to 1283 m above sea level (Table 1). The region experiences a semi-arid temperate continental monsoon climate that features a relatively fragile ecosystem, significant diurnal temperature variations, and a monthly mean temperature of 8.39 °C. The highest and lowest temperatures occur in July (22.96 °C) and December (−10.17 °C), respectively. Annual precipitation varies considerably, with rainfall in 2023 concentrated from June to September (the rainy season) accounting for 66.51%. The soil particle composition within the 0–120 cm profile comprises 1.65% clay particles, 12.10% silt particles, and 86.25% sand particles. The soil bulk density ranges from 1.49 to 1.62 g·cm^−3^ (Table 2), indicating a looser soil structure with poor water retention and increased susceptibility to wind and sand erosion. Over the past 50 years, a series of ecological restoration projects have been implemented, leading to substantial improvements in vegetation cover in the Mu Us Sandy Land [27]. The restored area in Uxin Banner spans 4.288.65 km^2^, accounting for 36.88% of the total area. Sandy plants dominate this region, and the primary shrub species are *A. ordosica*, *S. psammophila*, *C. korshinskii*, and *Sabina vulgaris*, etc.

### 3.2. Sample Collection and Processing

In this study, conducted from August 2023 to October 2023, Tuke Town, Uxing Banner, Ordos City, located in Mu Us Sandy Land, was selected to rely on the Key Laboratory of Aeolian Physics and Desertification Control Engineering from the Inner Mongolia Autonomous Region to conduct experiments on the correlation between hydrogen and oxygen isotopes and soil moisture. Sample plots were mainly selected from four silvicultural patterns (*Ao*, *Sp*, *Ao* × *Sp*, and *Ao* × *Ck*) that were artificially established in 1998. Near-ground-level aerial photographs were captured using a DJI Elf 4 drone to assess vegetation cover in the study area. Experimental areas with similar standing conditions, climatic variations, and >85% vegetation cover were selected (Figure 6). The key characteristics of each site including elevation, slope, slope direction, and vegetation growth status are listed in Table 1.

From August to October 2023, 27 rainfall events were monitored, totaling 147.5 mm, accounting for 42.29% of the annual precipitation. Eighteen of these events recorded <5 mm of rainfall, primarily occurring in September and October, whereas two events recorded >20 mm of rainfall, concentrated in August. The rainy season (August), transitional season (September), and dry season (October) were classified based on the amount of rainfall, contributing 62.51% (92.2 mm), 29.42% (43.4 mm), and 8.07% (11.9 mm) of the total precipitation during the study period, respectively.

**Figure 7:**
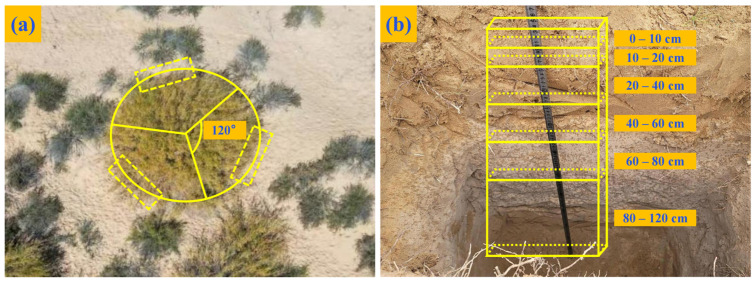
Sampling area selection: digging profiles around the base of tree (**a**). Post-excavation sampling profile (**b**). 0–10 cm indicates the depth of the soil layer 10 cm down from the soil surface, the rest of the content has this meaning.

During the test period, a homemade rainfall collection device was established around the sample site to collect rainwater in 50 mL sampling bottles after each downpour. A total of 36 rainwater samples were collected, sealed with Parafilm, and stored in a cryostat at −20 °C to analyze the stable isotopes of local precipitation. Three representative plants were selected from each pure forest sample plot on each sampling date, from August to October 2023. The profiles were obtained by locating the lateral roots of the trees in three directions using the base of the tree as the origin. An angle of 120° was maintained while selecting a section of the mixed forest sample plot situated between the two tree species and close to the root system following the aforementioned method (Figure 7a). The survey concluded that the root distribution of three typical sand-fixing vegetation types was primarily concentrated in *A. ordosica* (80 cm), *S. psammophila* (100 cm), and *C. korshinskii* (120 cm). Consequently, it was determined that the profile was excavated with dimensions of 0.8 m in length, 0.5 m in width, and 1.2 m in depth. Soil samples were collected from the bottom up at each soil depth interval (0–10, 10–20, 20–40, 40–60, 60–80, and 80–120 cm) within a specified depth range (Figure 7b).

Soil samples were collected from the bottom of each soil depth interval using a ring knife. A total of 216 ring knife samples were collected and transported to the laboratory for the analysis of soil moisture and other relevant indicators. The ring knife was placed in a flat-bottom tray filled with water to ensure flushing with the upper edge of the knife. After 12 h of water absorption, the weight of each sample was recorded as W_1_. A flat-bottomed tray containing dry sand was then added for 2 h to control the water content, and the weight was recorded as W_2_. Water control was continued for an additional 24 h, after which the weight was recorded as W_3_. Subsequently, the samples were dried in an oven at 105 °C for 24 h and the final weight was recorded as W_4_. The initial weight of the empty ring knife was denoted as W_0_, and the combined weight of the ring knife and the wet soil was recorded as W. The relevant indices were calculated based on these measurements [28].

Equations for soil moisture-related indicators:

(1) $$SWC=\frac{\left(W-W_{4}\right)}{W_{4}}\times 100\%$$

(2) $$BD=\frac{W_{4}-W_{0}}{V}$$

(3) $$FC=\frac{\left(W_{3}-W_{4}\right)}{W_{4}}\times 100\%$$

(4) $$WC=\frac{W-W_{4}}{W_{4}-W_{0}}$$

where *SWC* is the soil water content, *BD* is the soil bulk density, *FC* is the soil field capacity, *WC* is the wilting coefficient, and *V* is the ring knife volume (cm^3^).

At specified soil depth intervals, 216 soil samples were collected in glass sampling tubes with screw-caps. The tubes were sealed with Parafilm and stored in a cryostat at −20 °C for the determination of soil water stable isotopes. A small spade was used to collect 300 g of soil, which was then placed in a plastic bag. The 216 samples were then transported to the laboratory for natural drying. After removing impurities, the soil was passed through a 2 mm sieve for the analysis of soil mechanical composition using a laser particle sizer, and through a 0.149 mm sieve for the determination of soil organic carbon using the sulfuric acid digestion-potassium dichromate external heating method [29], as well as total nitrogen using the semi-micro-open-ended method [28].

On each sampling date, well-developed bolted standard branches in the canopy were pruned using high-pruning shears. Disease-free leaves from these branches were collected, mixed evenly, wrapped in aluminum foil, placed into 50 mL sampling tubes, sealed with Parafilm, stored in a cryostat at −20 °C, and transported to the laboratory for the analysis of stable isotopes in xylem water. Three parallel samples were established, yielding 54 samples.

### 3.3. Stable Isotope Analysis and Plant Water Source Identification

Soil and plant xylem water samples were extracted using an LI-2000 low-temperature vacuum distillation system (LICA, Beijing, China) [8]. The soil samples were pre-treated to remove large stones and other impurities. For the plant samples, the epidermis and unbolted green parts of the branches were removed before extraction. The processed samples were then placed in extraction tubes. To prevent the sample or fine particles from being ejected from the extraction tube due to air pressure during the extraction process, it was necessary to seal the opening of the extraction tube with degreasing cotton. After the samples were placed, the extraction system was evacuated using a vacuum pump. Extraction was initiated when the temperature of the extraction system reached 100 °C. Generally, the pumping durations for the soil and plant samples were 2.5 h and 3 h, respectively. Upon completion of the extraction, the moisture collected in the condenser tube was sealed with a Parafilm membrane, weighed, transferred to a 2 mL injection vial, and stored at a low temperature of 4 °C. The stable hydrogen and oxygen isotopic compositions of the liquid water (including soil water, plant xylem water, and rainwater) were determined using an L2130i Liquid Water Isotope Analyzer (LWIA) (Picarro, Santa Clara, CA, USA) [30]. The measurement accuracies were ±0.5‰ for δ^2^H and ±0.1‰ for δ^18^O. The ratios of the δ^2^H and δ^18^O values were calculated relative to the thousandth difference from the Standard Mean Oceanic Water. Data collected from precipitation samples and the stable hydrogen and oxygen isotopes of atmospheric precipitation from Uxin Banner, provided by the International Atomic Energy Agency, were used to analyze the relationship between soil water and precipitation.

The stable hydroxide isotope expression is as follows:

(5) $$\delta ({\%}_{{}^{\circ}})=\left(\frac{R_{sample}-R_{criterion}}{R_{criterion}}\right)\times 1000$$

where *R_sample_* and *R_criterion_* are the δ^2^H and δ^18^O abundances of the test sample and the international standard seawater, respectively.

Currently, research on vegetation water-use strategies primarily relies on three methods [31]: root distribution analysis [32], stem sap flow measurement, and stable hydrogen and oxygen isotope technology [33]. Although the analysis costs associated with the latter method are higher than those of the first two, they meet the accuracy and sensitivity requirements necessary for reliable research outcomes [34]. Consequently, it has become the predominant approach for studying vegetation water-use strategies. Research has demonstrated that isotopic fractionation typically does not occur in most vegetative root systems during water absorption until water reaches leaves or young unbolted branches. This finding provides a theoretical basis for using stable isotope techniques to clarify plant water-use strategies [35].

Because the research subject cannot directly utilize precipitation and groundwater and its root distribution is shallow, it is inferred that soil water is the sole source of vegetation growth in this study [36]. Hydrogen isotopes are significantly influenced by external factors compared with oxygen isotopes, and generally, there is no substantial difference in the distribution of hydrogen isotopes within the soil layer. Therefore, this study primarily used oxygen isotopes to analyze the sources of vegetation water use [37]. Two methods were used to determine the sources of the vegetation water utilization. First, a direct judgment method was used to identify the primary soil layer used by the vegetation. Typically, hydrogen and oxygen isotopes do not fractionate when the vegetation root system absorbs soil water [38]. Consequently, the source of vegetation water use can be predicted from the δ^18^O composition of the plant tissues. The δ^18^O values of plant water were compared with those of soil water, and the soil layers with the closest δ^18^O values were identified as the dominant sources of vegetation water use [39]. Although relying solely on stable isotope techniques could not accurately reflect the proportion of different water sources contributing to vegetation, these techniques were combined with modeling approaches to quantify the contribution values. Some researchers compared the model simulation results of MixSIAR, SIAR, MixSIR, and IsoSource, and found that the MixSIAR model yielded the best results for this analysis [40,41]. Therefore, the MixSIAR model was utilized in this study to differentiate and quantify the sources of water use from the rainy to dry seasons across various silvicultural patterns.

The MixSIAR model (version 3.1.12) accounts for potential uncertainties in the isotopic values of the mixture (plant xylem water) and the contributing source (soil water). It introduces the concept of a priori knowledge and demonstrates improved performance in differentiating water sources, enabling a more accurate analysis of the proportion of plant uptake of soil water from various soil layers. Principle of operation: Based on the principle of isotope mass conservation, the MixSIAR model was used to calculate the contributions of various soil layers to different forest stands, with a source increment set at 1% and mass balance tolerance set at 0.01. Because isotopic fractionation did not occur during plant water uptake, the discriminating values for δ^2^H and δ^18^O were set to 0 [33]. The run length of the Markov Chain Monte Carlo (MCMC) was designated as ‘long’ (chain length = 300,000; burn-in = 200,000; thinning = 100; chains = 3). MCMC was used to converge the posterior distributions of all the variables in the model. It was crucial to ascertain whether the model converged before accepting the output from the MixSIAR. The Gelman–Rubin and Geweke diagnostic tests were used to evaluate the convergence of the model [42]. ‘Residual only’ was specified as the error structure in the model, and an uninformative prior was established.

The δ^2^H and δ^18^O values of the different soil layers were entered into the model and calculated using the following equation:

(6) $$\begin{array}{l} \delta X=\sum_{i=1}^{n}ci\delta X_{i}\\ 1=\sum_{i=1}^{n}ci \end{array}$$

where *δX* is the δ^2^H and δ^18^O of plant stem water, *i* is the *i*th soil layer, *n* is the total number of soil layers. *δX*_i_ is the δ^2^H and δ^18^O of soil water in the *i*th soil layer, and *ci* is the contribution of the *i*th soil layer to vegetation.

The linear correlation lines consisting of δ^2^H and δ^18^O for regional precipitation and soil water were the local atmospheric precipitation line (LMWL) and soil water line, respectively. The linear offset (*lc–excess*) of different water bodies relative to the local atmospheric precipitation line δ^2^H as proposed by Landwehr and Coplen, is an indicator of the degree of evaporation [43]:

(7) $$lc-excess=\delta^{2}H-a\delta^{18}O-b$$

*A* and *b* are the slope and intercept of the LMWL, respectively, and δ^2^H and δ^18^O are the values of the hydroxide isotopes in the sample. When the water body δ^2^H–δ^18^O line is below the LMWL and l*c*–*excess* is <0, stable isotopes are enriched in the water body due to evaporation. When a positive value of *lc–excess* occurs in a water body, the water sample may have been influenced by sources other than precipitation (e.g., groundwater, river water, and lake water) [44].

Based on the variation in soil water content and isotopic composition, water from different soil layers (0–20, 20–60, and 60–120 cm) was categorized into three potential water sources. The three soil layers were determined as follows:

Shallow soils (0–20 cm): SWC (CV = 49%) and isotope ratios (CV = 20%) exhibited significant seasonal variations and were susceptible to evaporation and seasonal precipitation recharge [40].

Middle soil (20–60 cm): SWC (CV = 40%) and isotope ratios (CV = 19%) were less seasonally variable and influenced by rainwater infiltration [45].

Deep soil (60–120 cm): SWC (CV = 40%) and isotope ratio (CV = 17%) were small relative to those of the above two layers and could maintain a relatively stable state.

### 3.4. Data Analyses

Statistical analysis was performed using the SPSS software (version 26.0) to assess the normality of the data at a 95% confidence level. One-way analysis of variance was used to examine the changes in soil physicochemical properties, as well as hydrogen and oxygen isotopes, from the rainy season to the dry season at various soil depths for the same silvicultural pattern and at the same soil depth for different silvicultural patterns. The experimental data were summarized using Microsoft Excel 2021, and plots were generated using ArcMap 10.2 and Origin 2021.

## 4. Discussion

### 4.1. Hydrogen and Oxygen Isotope Variations Under Multifactorial Influences

**Figure 8:**
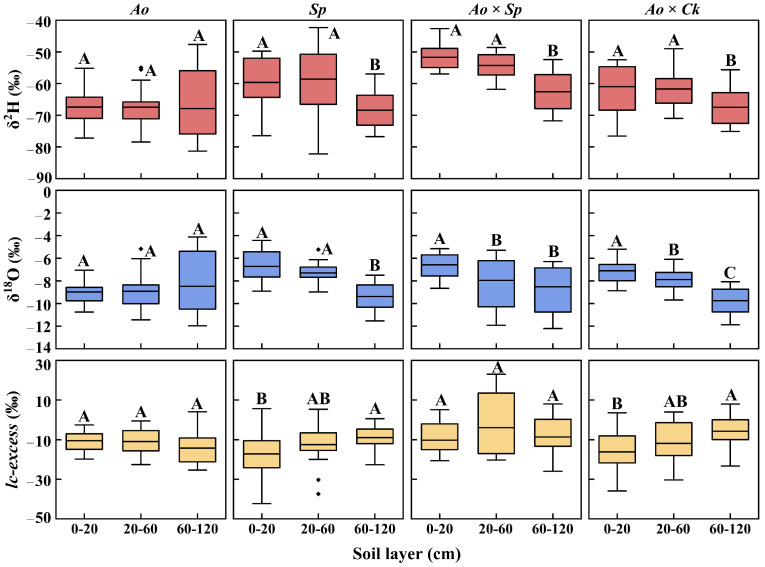
Box plots of stable isotope compositions (δ^2^H and δ^18^O) and lc-excess signature signals in the 0–20, 20–60, and 60–120 cm soil layers of four plantation forests during the study period. The *lc-excess* was calculated from the local atmospheric precipitation line (LMWL: δ^2^H = 6.39 δ^18^O + 0.56, *R*^2^ = 0.94, *n* = 36). The black spots around the error bar represent outliers. Capital letters on the boxes indicate significant differences between different soil depths under the same vegetation cover for each indicator (*p* < 0.05).

The disparities in regression slopes and intercepts for the soil water and xylem water lines across various plantation forests (Figure 2) were closely associated with factors such as water usage patterns and vegetation cover specific to each type of vegetation. Related research has shown that plant characteristics can disrupt the hydrological cycle, resulting in variations in the isotopic compositions of soil and xylem water among different stands [46]. Precipitation anomalies can induce pulsatile variations in the hydrogen and oxygen isotopes of soil water in dry and semi-arid regions characterized by fragile ecosystems [47]. Under the dual effects of differences in evaporation and infiltration processes and the mixing of old and new water from rainfall, the vertical gradients of stable isotopes all show a gradual depletion of isotopes with the deepening of the soil layer, and the same pattern of isotopic differences has been reported in previous studies [40]. The stable isotope signature of shallow soils predicts the precipitation signature and delineates evaporative fractionation resulting from rainfall [22]. The stable isotopes of soil water collected during the rainy season were significantly affected by precipitation, which was predominantly enriched in the upper soil layer and exhibited considerable variability. Before sampling during the transition period, the total rainfall was 20.6 mm, showing minor variations in stable isotope values compared with the rainy season, a pattern also revealed by previous studies [48]. The shift from the rainy season to the dry season is characterized by shallow enrichment and profound depletion. Some scholars have found, that is attributed to the shallow isotope values that are enriched compared to the deeper layers due to the more intense secondary evaporative fractionation of shallow soil water [49,50]. Anomalous variations in the δ^2^H and δ^18^O values of the *Ao* layer throughout the rainy season were tentatively hypothesized to correlate with distinct soil characteristics (such as grain size and porosity) associated with variations in vegetation types across the sample plots [51]. Relevant studies (Table 2) have indicated that the proportion of mechanical components in the *Ao* layer exhibits significant variability. Chalk particles dominate shallow soils, and after substantial precipitation events, chalk soils impede the infiltration of shallow soil water because of their particle size characteristics [52]. Soil water stable isotopes do not evaporate quickly and become enriched. Consequently, the δ^2^H and δ^18^O values of soil water appear to be enriched in the lower layers and impoverished in the upper layers. Li et al. conducted an isotope fractionation test on *A. ordosica* in the Mu Us Sandy Land and found that infiltration water formed from transitionally depleted water covered the profile above 60 cm, similar to the results of the present experimental study [53]. Except for the *Ao* layer, the remaining sample sites predominantly featured sandy soil (Table 2). Relevant studies indicate that soil moisture exhibits greater mobility among sandy grains characterized by low organic matter content and a loose soil structure, in contrast to clay and powder grains. These unique properties facilitate the rapid movement of water within sandy soils [54]. This characteristic adversely affects the selectivity of the soil water extraction and isotopic fractionation during water uptake [55]. Consequently, the stable isotopes of soil water in the remaining plantation forest showed shallow enrichment and layer-by-layer depletion with increasing soil depth (*p* < 0.05) (Figure 2), which again verified this property. Variations in evapotranspiration intensity across soil strata [56] resulted in a gradual decline in soil water δ^2^H and δ^18^O values with increasing soil depth, whereas the differences between pure and mixed forests diminished progressively (Figure 8). Except for *Sp*, the average values of δ^2^H and δ^18^O in shallow soil water across the woodlands were lower during the rainy season than during the dry season. This observation aligns with the findings of Pei Y et al. [57]. On the one hand, the dilution effect of the rainy season is indispensable [58], and on the other hand, the strong evapotranspiration effect of the dry season is quite influential [59]. The differing results observed in *Sp* were related to the varying residence times of water in the soil, which were influenced by the soil texture and root distribution (Table 2) [60]. It has been reported that the sparseness of the root system has a greater impact on water utilization than water uptake by deep-rooted vegetation [61], with denser root systems responding more quickly to sudden seasonal rainfall [62]. The important role of the root system in plant adaptation to drought has also been demonstrated by Jiang et al. in a related study [63]. Further investigation of the differences in soil texture under various vegetation types in the study area is necessary to fully substantiate these points.

Indicators characterizing evaporative fractionation of soil water were introduced to verify the accuracy of the aforementioned findings. The *lc-excess* value effectively predicted the strength of the evaporative fractionation signal in the soil water [64]. The transition from the rainy to dry season resulted in significantly negative *lc-excess* values in shallow soils, with higher values observed in mixed forests than in pure forests. This suggests robust evaporation from the soil surfaces of various plantations, with a distinct evaporation signal evident in pure forests [44] (Figure 8). During the rainy season, the *lc-excess* values were consistently negative (−16.82 ± 8.16‰) and showed no significant differences between pure and mixed forests. This indicates that stable isotopes were elevated owing to evapotranspiration during this phase [65]. Across all stands during the transition period, *lc-excess* values were higher in mixed forests than in pure forests (−4.71 ± 9.99‰), with positive *lc-excess* values noted in the middle and deep soils of the mixed forests. This suggests that this phase may be influenced by water sources beyond precipitation, such as groundwater, river water, and lake water [44]. It has also been found that the lag in water acquisition by *S. psammophila* leads to reliance on small amounts of groundwater [60]. The *lc-excess* values of different plantations during the dry season were higher in pure forests than in mixed forests (−11.31 ± 7.51‰), indicating that evapotranspiration was more pronounced in the mixed forests, which is consistent with the stable isotope reflections of soil water. In this study, only the effect of precipitation was considered when examining soil water use characteristics. However, when precipitation fails to meet the soil water supply, vegetation can exhibit a significant dependence on alternative water sources, such as groundwater [66]. Consequently, it is essential to incorporate the analysis of stable isotopes from various water sources, including groundwater, to quantitatively assess the impact of these sources on the water-use characteristics of vegetation in mixed forests and improve the identification of water sources utilized by vegetation.

### 4.2. Vegetation Water Utilization Strategies Under Coupled Two-Factor Disturbances

**Figure 9:**
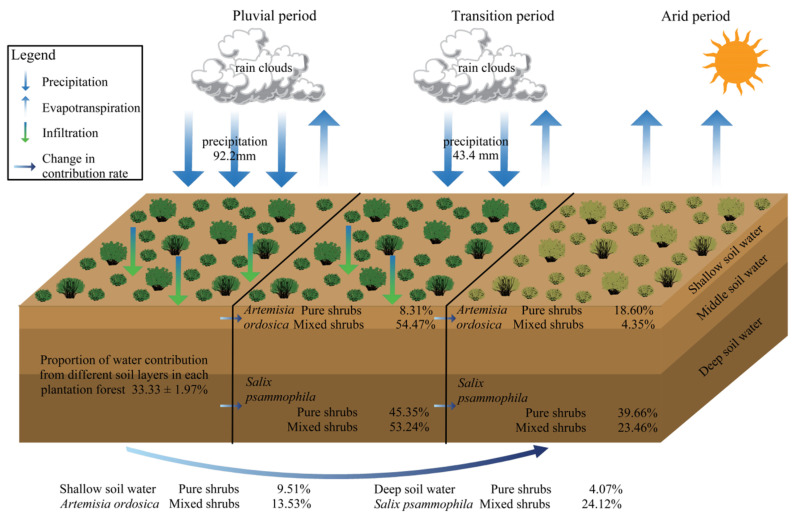
Change in the contribution ratio of vegetation water use in the main water-absorbing soil layer from the rainy season to the dry season.

Vegetation water-use strategies are influenced by arid environments and vegetation types in various ways (Figure 4 and Figure 5) [24]. Soil moisture is affected by precipitation, and the spatial heterogeneity of soil moisture changes, leading to a shift in the sources of water utilized by vegetation [48]. Different vegetation types determine primary water sources based on their root distribution and interspecific relationships [8]. Vegetation water use strategies adapt to changes in precipitation conditions [67]. Vegetation water use sources of *S. psammophila* and *C. korshinskii* species gradually migrate from shallow to deep soils during the rainy to the dry season transition, and other studies have shown similar results [37,68]. This phenomenon is collectively referred to as the dimorphic structural characteristics of the vegetative root system [8,69,70]. The minor variations in water source utilization observed in *A. ordosica* plants in response to changes in water conditions indicate a low level of plasticity in their water usage [40]. When moisture conditions were consistent in the study area (Figure 9), the mixed forest increased the proportion of water use across each primary soil layer, while reducing water consumption in the remaining layers compared with the water utilization patterns observed in pure forests. This is related to the nature of the soil in mixed forests, and the related experiment was carried out to find that the field water holding capacity of mixed forests was significantly higher than that of pure forests (*p* < 0.05) (Table 2). This difference may result in elevated water storage in mixed shrubs compared to pure shrubs, leading to an elevated proportion of water uptake in mixed shrubs [71]. During periods of abundant precipitation during the rainy season, the soil water content increased at all studied locations. The water supply from various soil layers to vegetation showed no significant differences across the wooded areas. Similar results were noted at the southern periphery of the Mu Us Sandy Land [70], where the distribution of soil moisture surpassed interspecific interactions as the primary regulatory mechanism governing the water supply to vegetation when the soil was sufficiently hydrated [72]. Throughout the transition period, disparities in water utilization by vegetation became evident. *A. ordosica* predominantly accessed medium and deep soil water (20–120 cm) in pure forests, whereas in mixed forests, it primarily utilized shallow soil water, exhibiting an increase of >30% compared with pure forests. The main soil layer utilized by *A. ordosica* shifted, which was related to the soil texture of the sampling site [54] and its adaptation to interspecies coexistence in mixed forests, allowing for a self-regulated depth of water utilization [73]. Undisturbed by precipitation during the dry season, the source of vegetation water use in each woodland area was strongly influenced by the root distribution. Most vegetation adapts to drought stress, primarily by absorbing soil water through the main root system [74]. The primary water-absorbing soil layer for *A. ordosica*, whether in pure or mixed forests, is located in the superficial soil layer and is closely associated with the dispersion of its root system. Several studies have investigated the root distribution of *A. ordosica* near the study area. They found that the root system predominantly occupied the superficial soil layer, with coarse roots primarily located in the 0–20 cm soil layer (81%) and fine roots in the 0–30 cm soil layer (79%) [75]. Comparable findings were observed in the present study during the excavation of the sampling profiles. Similar to *A. ordosica*, *S. psammophila* exhibited analogous outcomes and principles. Compared with pure forests, the overall enhancement rate of water utilization in dry-season mixed forests decreased by 50% during the transition period. This phenomenon was initially hypothesized to be related to reduced soil water availability resulting from reduced water recharge in the study area, as well as the complex distribution of belowground root systems in mixed forests, which leads to varying degrees of increased water consumption across different soil layers (Figure 9). Ultimately, it follows the rule of change that vegetation with different root distributions shows different ecological statuses of water absorption, i.e., shallow-rooted vegetation absorbs shallow soil water and deep-rooted vegetation absorbs deep soil water [76,77]. In this study, the depth of the root distribution was observed and recorded at the time of profile excavation. A more detailed investigation of the root system, considering factors, such as root length, density, and biomass, could be conducted to clarify the interactions between root system depth and water-use efficiency under different precipitation patterns [23].

Overall, the methods of water use by vegetation are influenced by arid environments and various vegetation types, resulting in variability in water use among species and promoting interspecific coexistence [23]. Under varying moisture conditions, vegetation primarily relies on shallow soil water during the rainy season and gradually transitions to areas with denser vegetation and deeper root growth as rainfall decreases. Mixed forests enhance water utilization when the vegetation types differ. These two factors indicate that drought conditions inhibit the ability of mixed forests to enhance water utilization compared with pure forests.

### 4.3. Revelation of Vegetation Water Utilization Strategies Under Integrated Consideration of Water Conditions and Configuration Patterns

Understanding the various patterns of water consumption by vegetation is crucial for effectively managing available water resources and optimizing the use of local flora. It is vital to achieve logical advantages and to ensure the sustainability of vegetation restoration initiatives [3]. During the rainy season and the subsequent transition period in the study area, water use efficiency under the mixed vegetation pattern demonstrated a significant enhancement compared with the pure forest. However, the promotion effect somewhat diminished owing to the decrease in rainfall. This observation highlights the feedback of water-use strategies in response to changes in arid environments and vegetation configurations. Based on the findings of this study, we provided detailed recommendations for the construction and management of vegetation restoration and afforestation projects. The two hybrids examined in this study exhibited strong partitioning between species, which was attributed to their root distribution and water-uptake patterns. These characteristics make them particularly suitable for hybridization, considering local water resources, and they can be implemented on a larger scale during subsequent phases of vegetation development in arid and semi-arid regions. Priority should be given to drought-resistant and low-water-consuming vegetation with traits similar to those identified in the present study. However, careful planning of plantation forest construction is essential to adjust the proportions of hybrid species in mixed forests and to optimize planting density according to local conditions. Additionally, it may be beneficial to replace the shrub and bush mixture with a blend of shrubs and herbs, which would enhance the wind and sand control capabilities of the region. The role of herbaceous plants in the conservation of shallow soil water can provide significant ecological benefits. Maximize the advantages of shrubs and herbs to promote vegetation root growth [78]. Take advantage of the intricate entanglement and consolidation of the root system in the soil to increase soil porosity and accelerate the infiltration and storage of soil moisture to ensure that the water retention capacity of the local soil is solidly enhanced [79]. In the context of unstable climate change, measures such as drought relief and increasing the frequency of irrigation can be taken in advance when extreme weather is monitored to enhance the efficiency of vegetation water use and ecological stability. By applying reasonable and appropriate amounts of watering measures, we can improve the water utilization of vegetation, thereby preventing issues such as heat stress, drought, large-scale plant die-off, and excessive irrigation, which lead to water resource waste and soil salinization. Additionally, incorporating water-saving irrigation techniques such as drip and sprinkler irrigation can further enhance the efficiency of irrigation water use.

In summary, future research could include the following points based on this experiment: (1) Expanding the study of vegetation water utilization across various planting densities while comprehensively considering water utilization strategies at these densities. (2) Conducting in-depth research on soil moisture and exploring multiple water sources such as groundwater and lake water to clarify how different water sources respond to vegetation water use [37]. (3) Investigate vegetation root systems to further identify the factors influencing biases in vegetation water-use sources [40]. (4) Focusing on the species scale, conducting tests on the dynamic density changes of shrubs in relation to local moisture throughout their life cycle [20], and elucidating the patterns of change in response to seasonal climate variations and other factors. The management measures and future research directions proposed in this study can serve as a reference for artificial revegetation efforts in other arid and semi-arid regions with similar environmental conditions and vegetation-related water-use strategies.

## 5. Conclusions

This study combined stable isotope techniques with MixSIAR modeling to provide novel insights into the water-use patterns of typical pure and mixed shrubs under changing moisture conditions in the Mu Us Sandy Land hinterland. Arid environments and vegetation configuration patterns significantly influenced vegetation water use strategies. Under varying moisture conditions, as rainfall amounts decreased, the isotopic values of soil water, δ^2^H (−61.77 ± 8.61‰) and δ^18^O (−8.13 ± 1.82‰) gradually became depleted. Except for the small shrub *A. ordosica*, the water utilization sources of other vegetation types shifted from shallow to primarily deep soil water, indicating a strong ecological plasticity driven by root systems. When vegetation configuration patterns varied, the stable isotope values of soil water in mixed shrubs (δ^2^H: −59.79 ± 7.66‰, δ^18^O: −7.96 ± 1.81‰) were more enriched compared with those in pure shrubs (δ^2^H: −64.90 ± 9.25‰, δ^18^O: −8.30 ± 1.80‰). Additionally, mixed shrubs exhibited a specific enhancing effect on the proportion of soil water utilized by plants compared with pure shrubs. When considering both factors simultaneously, as rainfall decreased, the positive impact of mixed shrubs on water utilization compared with pure shrubs gradually decreased, with the maximum increase rate fluctuating from 39.53% down to 12.66%. Ultimately, it was concluded that vegetation’s proportion of soil water uptake in the main water supply soil layer is significantly enhanced in brief drought environments compared to the rainy season. This enhancement is suppressed after a prolonged drought. Using this mechanism, we can select appropriate vegetation types, strategically plan their configuration, and enhance vegetation water management for future vegetation construction and management efforts. This approach provides valuable references for the efficient use of water and scientific delineation of water resources in similar regions. It is of great significance to improve strategies for vegetation water utilization in arid and semi-arid zones as well as for planning the development of plantation forests.

## Figures and Tables

**Table 1:** Essential characteristics of sample plot.

Configuration Mode	L_l_	S	A (m)	S_d_ (hm^2^)	T_h_ (m)		C (m)	B_d_ (cm)
*Ao* (pure)	38°53′18.36″ E 109°11′48.58″ N	2°	1275	3767	0.62 ± 0.14		0.86 ± 0.32	0.21 ± 0.41
*Sp* (pure)	38°59′15.84″ E 109°21′24.56″ N	5°	1278	733	2.27 ± 0.31		2.06 ± 0.47	0.09 ± 0.02
*Ao* × *Sp* (mixed)	38°59′10.41″ E 109°21′31.21″ N	4°	1281	2300 (1:8)	Mixed *Ao*	0.78 ± 0.13	1.18 ± 0.27	0.06 ± 0.03
					Mixed *Sp*	3.17 ± 0.80	3.23 ± 1.03	0.11 ± 0.03
*Ao* × *Ck* (mixed)	39°1′42.29″ E 109°23′10.06″ N	2°	1283	2500 (1:7)	Mixed *Ao*	0.70 ± 0.16	1.16 ± 0.46	0.08 ± 0.04
					Mixed *Ck*	2.12 ± 0.44	2.80 ± 0.52	0.11 ± 0.04

*Ao*, *Sp*, *Ao* × *Sp*, and *Ao* × *Ck* denote pure stands of *A. ordosica*, pure stands of *S. psammophila*, mixed stands of *A. ordosica* and *S. psammophila*, and mixed stands of *A. ordosica* and *C. korshinskii*, respectively. Abbreviated letters are used throughout. Longitude & Latitude (L_l_), Slope (S), Altitude (A), Stand density (S_d_), Tree height (T_h_), Crown (C), and Basal diameter (B_d_).

**Table 2:** Basic physical and chemical properties of soil under different plantation forest cover types.

Configuration Mode	Soil Layer (cm)	SOC (g·kg^−1^)	TN (g·kg^−1^)	Soil Mechanical Composition (v%)			BD (g·cm^−3^)	FC (v%)	WC (v%)
				Clay	Particle	Sand			
*Ao* (pure)	0–20	5.67 ± 0.25 Ab	0.53 ± 0.01 Aa	5.02 ± 0.31 Aa	61.33 ± 4.04 Aa	33.65 ± 4.31 Cc	1.53 ± 0.33 Aab	5.15 ± 0.11 Ac	4.56 ± 0.45 Aa
	20–60	3.19 ± 0.67 Ba	0.26 ± 0.002 Ba	2.63 ± 0.49 Ba	15.74 ± 2.77 Ba	81.64 ± 3.25 Bb	1.53 ± 0.01 Aa	2.58 ± 0.10 Bc	1.55 ± 0.19 Bb
	60–120	1.18 ± 0.13 Cab	0.14 ± 0.01 Cb	1.58 ± 0.05 Ca	7.78 ± 0.22 Cb	90.64 ± 0.27 Ab	1.55 ± 0.06 Aa	2.10 ± 0.12 Cc	1.62 ± 0.08 Bb
*Sp* (pure)	0–20	3.01 ± 0.45 Ac	0.34 ± 0.01 Ab	1.88 ± 0.35 Ab	13.12 ± 2.44 Ab	85.00 ± 2.79 Bb	1.54 ± 0.05 Aab	3.72 ± 0.23 Ad	0.99 ± 0.08 Bc
	20–60	1.84 ± 0.47 Bb	0.15 ± 0.01 Cb	1.15 ± 0.06 Bb	6.52 ± 0.38 Bb	92.32 ± 0.43 Aa	1.56 ± 0.05 Aa	2.48 ± 0.32 Bc	0.74 ± 0.08 Bc
	60–120	1.57 ± 0.36 Ba	0.23 ± 0.0005 Ba	1.52 ± 0.14 ABa	15.48 ± 1.42 Aa	83.00 ± 1.56 Bc	1.58 ± 0.09 Aa	2.11 ± 0.19 Bc	3.56 ± 0.72 Aa
*Ao* × *Sp* (mixed)	0–20	6.33 ± 0.40 Aa	0.19 ± 0.003 Ac	1.29 ± 0.03 Ac	6.07 ± 0.11 Ac	92.65 ± 0.13 Ba	1.49 ± 0.08 Bb	7.40 ± 0.34 Ab	2.00 ± 0.15 Ab
	20–60	1.67 ± 0.63 Bb	0.12 ± 0.003 Bc	0.95 ± 0.11 Bb	4.17 ± 0.48 Bb	94.88 ± 0.59 Aa	1.56 ± 0.02 ABa	5.78 ± 0.15 Bb	1.28 ± 0.29 Bb
	60–120	1.51 ± 0.04 Ba	0.09 ± 0.01 Cc	1.04 ± 0.13 Bb	4.31 ± 0.48 Bc	94.66 ± 0.61 Aa	1.62 ± 0.06 Aa	4.33 ± 0.15 Cb	1.13 ± 0.12 Bb
*Ao* × *Ck* (mixed)	0–20	1.47 ± 0.23 Ad	0.13 ± 0.007 Bd	1.36 ± 0.16 Ac	6.30 ± 0.78 Ac	92.35 ± 0.93 Ba	1.62 ± 0.01 Aa	9.37 ± 0.50 Aa	1.98 ± 0.31 ABb
	20–60	1.56 ± 0.54 Ab	0.15 ± 0.01 Ab	1.19 ± 0.12 ABb	5.20 ± 0.47 Ab	93.61 ± 0.59 Ba	1.58 ± 0.09 Aa	9.07 ± 0.58 Aa	2.53 ± 0.19 Aa
	60–120	0.83 ± 0.18 Ab	0.04 ± 0.01 Cd	0.95 ± 0.09 Bb	3.95 ± 0.28 Bc	95.10 ± 0.37 Aa	1.60 ± 0.05 Aa	6.86 ± 0.51 Ba	1.49 ± 0.35 Bb

SOC, TN, BD, FC, and WC are organic matter, total nitrogen, bulk density, field capacity, and wilting coefficient, respectively. Capital letters indicate significant differences between the soil depths under the same vegetation cover (*p* < 0.05). Lowercase letters indicate significant differences between vegetation cover at the same soil depth (*p* < 0.05), (mean ± standard deviation [SD], *n* = 3).

## Data Availability

All the data supporting the conclusions of this article are included in this article.
